# Alpha-Synuclein Lesions in the Peripheral Nervous System of the Larynx in Parkinson’s Disease

**DOI:** 10.1007/s00455-025-10870-y

**Published:** 2025-08-23

**Authors:** Liancai Mu, Jingming Chen, Themba Nyirenda, Jing Li, Karen Wheeler Hegland, Charles H. Adler, John N. Caviness, Holly A. Shill, Geidy E. Serrano, Thomas G. Beach, The Arizona Parkinson’s Disease Consortium

**Affiliations:** 1 Upper Airway Research Laboratory, Center for Discovery and Innovation, Hackensack Meridian Health, 111 Ideation Way, Nutley, NJ 07110 USA; 2 Upper Airway Dysfunction Laboratory, Department of Speech, Language and Hearing Sciences, College of Public Health and Health Professions, University of Florida, 1225 Center Dr., Gainesville, FL 32611 USA; 3 Department of Neurology, Mayo Clinic Arizona, 13400 E. Shea Blvd, Scottsdale, AZ 85259 USA; 4 Barrow Neurological Institute, 2910 N 3rd Ave, Phoenix, AZ 85013 USA; 5 Banner Sun Health Research Institute, 10515 West Santa Fe Dr, Sun City, AZ 85351 USA

**Keywords:** Parkinson’s disease, Larynx, Alpha-synuclein, Swallowing, speech and voice disorders

## Abstract

Swallowing, speech and voice (SSV) disorders are very common in Parkinson’s disease (PD). The aim of the present studies was to test our hypothesis that PD pathology affects the peripheral nervous system (PNS) of the larynx, thus possibly contributing to SSV deficits. Twenty-eight adult human larynges obtained from autopsied subjects with clinically diagnosed and neuropathologically confirmed PD (n = 20) and age-matched healthy controls (n = 8) were studied. Three laryngeal nerves (i.e., recurrent laryngeal nerve, RLN; external and internal superior laryngeal nerves, ESLN and ISLN), three muscles (i.e., thyroarytenoid, TA; posterior cricoarytenoid, PCA; and cricothyroid, CT), and three mucosa samples overlying the larynx and laryngopharynx (i.e., true vocal fold, TVF; laryngeal surface of the epiglottis, LSE; and aryepiglottic fold, AEF) were examined to detect phosphorylated α-synuclein (PAS) aggregates, the pathological hallmark of PD. The severity of the PAS lesions in the examined tissues was quantified by using a total PNS pathology score we newly developed. The results showed that the larynx was affected by PAS pathology in PD subjects but in none of the controls. The relative contributions of the PNS and brain pathologies to SSV disorders were analyzed. In this series, SSV severity levels in a substantial percentage (45%) of PD patients were more consistent with PNS than brain pathology severity levels. These findings suggest that in addition to brain pathology, PAS lesions in the PNS of the larynx also play an important role in the development of SSV disorders in PD.

## Introduction

The larynx is an important structure for life-maintaining functions such as breathing, phonation, swallowing, and airway protection. These functions are affected by a variety of neurologic disorders, including Parkinson's disease (PD). The majority of individuals with PD have swallowing, speech and voice (SSV) disorders [for review, see 1]. It has been reported that 50–80% of PD patients have dysphagia and 90% have impaired voice and speech [1–3]. Dysphagia can result in malnutrition, dehydration, and life-threatening conditions such as choking, aspiration pneumonia, and death [4–7]. Up to 70% of patients with PD die from aspiration pneumonia [6, 8, 9]. Voice and speech disorders (i.e., dysphonia and dysarthria) are particularly disabling conditions that influence social interactions and alter activities of daily living [1–3].

PD was long considered to be a central nervous system (CNS) disorder caused by the death of dopamine-producing neurons in the substantia nigra [10]. This death of neurons is related to the presence of intracellular inclusions known as Lewy bodies and Lewy neuritis, which are primarily composed of an aggregated form of the protein α-synuclein. Aggregates of phosphorylated α-synuclein (PAS) underlie PD pathology [for review, see 11, 12].

SSV disorders in PD are traditionally believed to be associated with the same dopaminergic deficiency as that causing limb bradykinesis and rigidity [13, 14]; however, there is little evidence to support this. For example, severity of SSV deficits in PD does not corelate well with overall muscle rigidity score and disease severity [15–17]. Moreover, SSV disorders generally have poor responses to anti-PD treatments that significantly improve limb motor function [1–3, 18, 19]. These clinical observations suggest that SSV disorders may be not solely related to nigrostriatal dopamine deficiency. Instead, other neurotransmitter systems may be involved and degeneration of these systems may contribute to SSV disorders [15, 18, 20].

Additionally, PD pathology has been identified not only in the brain but also in the peripheral nervous system (PNS). Therefore, PD is currently appreciated as a broader disease affecting many areas of the nervous system, including the autonomic nervous system [for reviews, see 1, 21] and PNS of the upper aerodigestive tract [22–25]. Our recent studies provided the first demonstration of PAS lesions in the pharyngeal motor [22] and sensory [23] nerves, muscles [24], and mucosa [25] from PD subjects. Notably, PD patients with dysphagia had higher density of PAS lesions in the pharyngeal nerves as compared with those without dysphagia. These findings suggest that PAS pathology in the PNS controlling the upper aerodigestive tract contributes, at least in part, to SSV disorders in PD. Although laryngeal dysfunction is associated with SSV disorders in PD, no study to date has looked at the PD larynx.

This study was designed to detect PAS lesions in the PD larynx, identify possible correlations of the severity of SSV disorders with PNS and brain PAS severity, and estimate the relative contributions of PNS and CNS pathologies to the SSV deficits to test our central hypothesis that SSV disorders in PD patients are caused not only by nigrostriatal dopamine deficiency but also by PAS pathology in the PNS controlling the structures in the upper aerodigestive tract, including the larynx, pharynx, and tongue.

## Materials and Methods

### Human Subjects and Tissue Source

In our prior studies on PD pharynx [22–25], 20 whole-mount (larynx-pharynx-tongue-soft palate) specimens and psoas major muscles (control) were obtained from autopsied subjects with clinically diagnosed and neuropathologically confirmed PD. In the present study, the larynges from the 20 whole-mount PD specimens and 8 age-matched healthy controls were used to detect the abnormalities that may exist in the laryngeal nerves, muscles and mucosa. The PD specimens were provided by the Arizona Study of Aging and Neurodegenerative Disorders (AZSAND) and the Brain and Body Donation Program (BBDP) [26] at Banner Sun Health Research Institute. The healthy autopsied specimens without known systemic neuromuscular disorders were obtained from Department of Pathology at Mount Sinai Medical Center in New York City.

### Inclusion/Exclusion Criteria for Specimen Procurement

We developed an inclusion/exclusion plan for specimen procurement. Specimens meeting the criteria as described below were accepted. Inclusion criteria for PD specimens were defined as follows: (1) The PD specimens from the donors must be clinicopathologically diagnosed as PD; (2) PD subjects with or without SSV disorders were included; (3) Ages ranging from 55 to 85 were included; and (4) No ethnic backgrounds were excluded. Exclusionary criteria for PD specimens included: (1) cases without an established diagnosis of PD; (2) parkinsonian symptoms caused by other diseases; (3) SSV disorders caused by neurological disorders other than PD; and (4) other confounding conditions such as a history of malignancy, local trauma, surgery, radiotherapy or chemotherapy at the head and neck region. Age-matched controls were selected only when the subjects had no neuromuscular disorders affecting functions of the upper aerodigestive tract and fulfilled the exclusion criteria.

### PD and SSV Evaluations

The AZSAND/BBDP provided detailed clinical and neuropathological data for each of the autopsied PD subjects (Table 1). All PD patients received annual standardized cognitive testing (neuropsychological test battery) and movement neurological examinations. Disease severity was clinically rated using the Hoehn and Yahr (H&Y) Scale [27] and disability and motor impairment using the Unified Parkinson’s Disease Rating Scale (UPDRS) [28]. Specific clinicopathological diagnostic criteria for PD were used [29].

**Table 1:** Demographic, clinical features, and PNS and CNS pathology severity in PD subjects and normal controls

Case no.	Sex	Age at death, years	Age at PD onset, years	PD duration, years	Last H&Y stages	Last motor UPDRS	UPDRS months before death	PMI, hours	Swallowing Score (0–4)	Speech Score (0–4)	TPPS (0–3)	TBS (0–40)	SNPNL Score (0–3)
PD 1	M	75	55	20	2	17	21	69	1	2	1	35	3
2	M	73	62	11	3	18	26	26	0	3	2	37	3
3	M	78	59	19	4	51	8	76	2	2	1	–	–
4	F	84	64	20	3	29	10	48	0	1	1	26	3
5	M	80	69	11	4	53	11	26	0	3	2	37	2
6	M	81	70	11	4	43	19	58	1	0	1	30	3
7	F	79	68	11	4	47	9	16	0	2	1	36	2
8	M	75	45	30	4	66	1	36	2	4	3	35	3
9	M	80	63	17	5	40	5	23	0	3	3	–	2
10	M	79	56	23	2	28	9	34	2	3	3	26	2
11	M	76	66	10	4	55	3	50	2	4	3	36	2
12	M	69	64	5	5	54	1.5	56	4	4	3	24	3
13	M	74	53	21	4	66	10	24	1	4	3	28	3
14	M	73	67	6	2	41	5	50	1	0	1	27	3
15	M	69	54	15	4	45	4.5	29	1	3	2	29	3
16	F	77	48	29	3	20	28	23	2	1	1	–	2
17	M	77	72	5	3	52	8	84	1	2	2	27	3
18	M	74	68	6	2	21	15	34	1	2	1	29	3
19	M	76	64	12	4	31	10	34	2	3	3	30	3
20	F	65	58	7	5	68	18	28	2	3	2	36	3
Mean (range)		**76** (65–84)	**61** (45–72)	**15** (5–30)	**3.6** (2–5)	**42** (17–68)	**11** (1–28)	**41** (16–84)	**1.3** (0–4)	**2.5** (0–4)	**2.0** (1–3)	**31** (24–37)	**2.7** (2–3)
NC 1	F	74	–	–	–	–	–	24	–	–	–	–	–
2	M	80	–	–	–	–	–	73	–	–	–	–	–
3	M	70	–	–	–	–	–	26	–	–	–	–	–
4	F	70	–	–	–	–	–	30	–	–	–	–	–
5	F	78	–	–	–	–	–	28	–	–	–	–	–
6	M	76	–	–	–	–	–	32	–	–	–	–	–
7	F	78	–	–	–	–	–	40	–	–	–	–	–
8	M	78	–	–	–	–	–	42	–	–	–	–	–
Mean (range)		**76** (70–80)						**37** (24–73)					

CNS, central nervous system; F, female; H&Y stages, Hoehn and Yahr clinical rating scale (range, 1–5); M, male; NC, normal control; PD, Parkinson’s disease; PMI, postmortem interval; PNS, peripheral nervous system; SNPNL, substantia nigra pigmented neuron loss; TBS, total brain score; TPPS, total PNS pathology score; UPDRS, Unified Parkinson’s Disease Rating Scale

SSV impairments in PD patients were assessed subjectively using UPDRS ratings (i.e., swallowing and speech scores) (Table 1). A swallowing score (0–4) was given by using item 7 of the UPDRS Part II Scale (i.e., swallowing score of 0 = normal; 1 = rare choking; 2 = occasional choking; 3 = requires soft food; and 4 = requires nasogastric tube or percutaneous endoscopic gastrostomy feeding). A speech score (0–4) was given by using items 5 and 18 of the UPDRS Parts II and III Scales (i.e., speech score of 0 = normal; 1 = mildly affected, no difficulty being understood, slight loss of expression, diction and/or volume; 2 = moderately impaired, monotone, slurred but understandable; 3 = marked impairment, difficult to understand; 4 = unintelligible). In this study, several PD patients also received some objective SSV assessments, including modified barium swallow (MBS)—performed by radiologists with speech pathologist assistance—and fiberoptic flexible laryngoscopy or laryngeal videostroboscopy performed by otolaryngologists.

### Brain Neuropathologic Assessments

One of the objectives of this study was to determine the relative contributions of PNS vs. CNS pathology to SSV disorders in PD. In this series, all PD subjects received brain neuropathologic assessments after death. Brain pathology severity was rated by an experienced neuropathologist (Dr. Thomas G. Beach), who provided a detailed neuropathologic report for each subject. Specifically, all autopsied PD cases had standardized brain PAS semi-quantitative density grading in 10 standard brain regions (total possible score of 40) (Table 1) and staging of brain PAS distribution using the Unified Staging System for Lewy Body disorders (USSLB) developed by Beach et al. [30]. The reliability and validity of the USSLB has been confirmed by subsequent independent studies [31–33]. For statistical purposes, semi-quantitative microscopic lesion density estimates were converted to numerical scores from 0 to 4 for Lewy-type α-synucleinopathy. For correlational analyses, the PAS density scores of the 10 brain regions are summed to a single global score for each subject. In addition, all autopsy cases received a substantia nigra pigmented neuron loss (SNPNL) score (Table 1), a measure of CNS dopamine depletion for correlation analyses. The SNPNL score is a semi-quantitative score (0–3) that has been validated by strong and significant correlation with striatal tyrosine hydroxylase ELISA [30].

### Laryngeal Tissue Sampling and Preparation

The larynx specimens were obtained from 1 to 2 days after death (Table 1); this postmortem interval does not hinder reliable morphological and histochemical analysis of autopsied tissues when the body has been stored in a refrigerated area [22–25, 34–37].

Each larynx was bisected in the midline, forming a left and a right specimen. In this study, the left semi-larynges from PD subjects 1, 3, 5, 7, 9, 11, 13, 15, 17, 19 and right semi-larynges from PD subjects 2, 4, 6, 8, 10, 12, 14, 16, 18, 20 were investigated. For normal control (NC) group, left semi-larynges from NC 1, 3, 5, 7 and right semi-larynges from NC 2, 4, 6, 8 were studied.

**Fig. 1:**
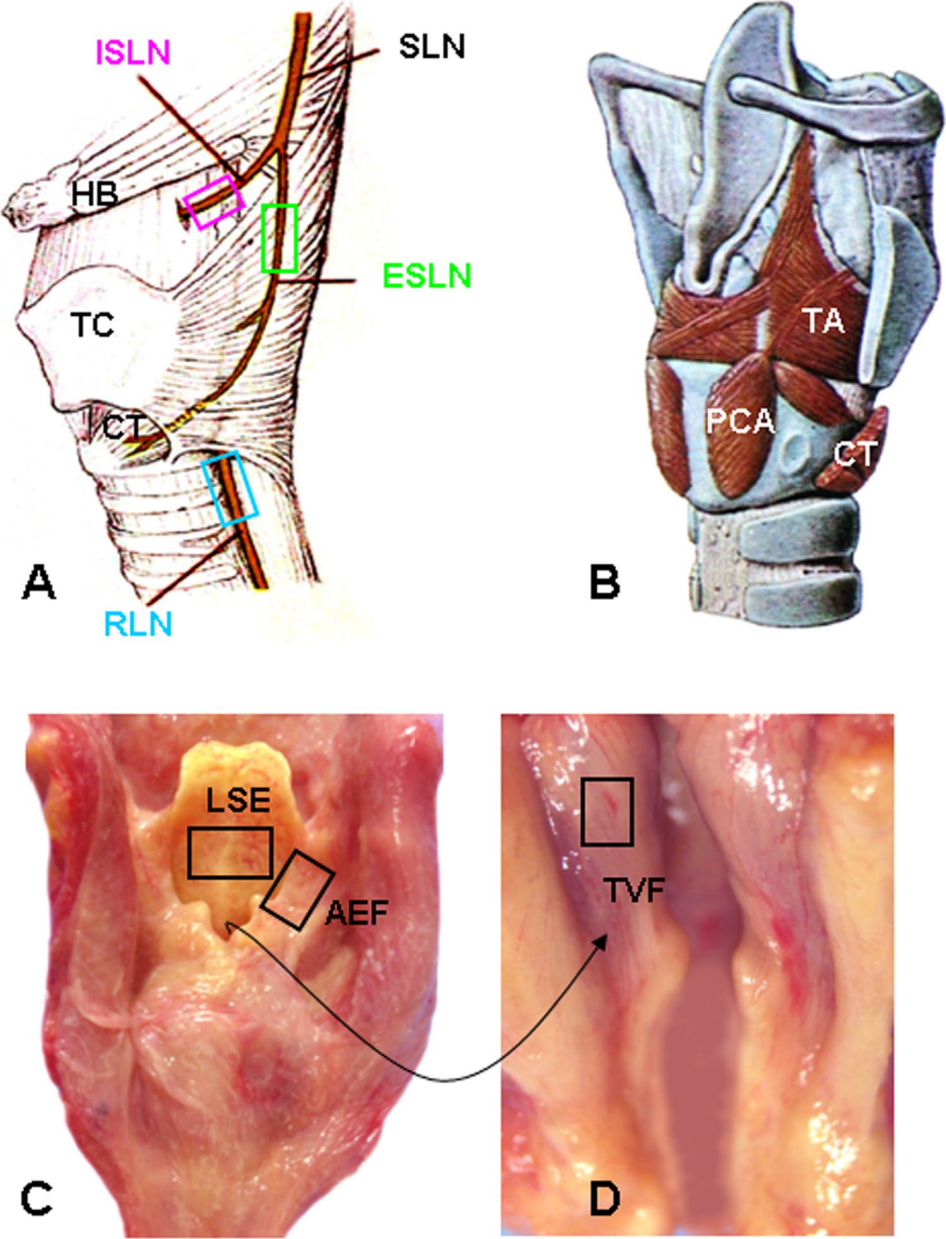
Schematics and photographs, showing sampling sites of the laryngeal nerves (boxed regions in (**A**), muscles (**B**), and mucosa (boxed regions in **C** and **D**). The photograph from inside of the larynx (**D**) shows the sampling site of the mucosa overlaying the true vocal fold (TVF). AEF, aryepiglottic fold; CT, cricothyroid muscle; ESLN, external superior laryngeal nerve; HB, hyoid bone; ISLN, internal superior laryngeal nerve; LSE, laryngeal surface of the epiglottis; PCA, posterior cricoarytenoid muscle; RLN, recurrent laryngeal nerve; SLN, superior laryngeal nerve; TA, thyroarytenoid muscle; TC, thyroid cartilage

For each larynx, the studied tissues included nerves, muscles, and mucosa. The nerves included recurrent laryngeal nerve (RLN), external superior laryngeal nerve (ESLN), and internal superior laryngeal nerve (ISLN). A 10-mm-long segment of each nerve was sampled before they enter the larynx (Fig. 1A), fixed with 10% neutral buffered formalin overnight, sectioned longitudinally (5 µm thick), and prepared for PAS immunohistochemistry. Three muscle samples (10 × 10 mm/each) were taken from RLN-innervated thyroarytenoid (TA; adductor of the vocal fold) and posterior cricoarytenoid (PCA; abductor of the vocal fold) muscles, and ESLN-innervated cricothyroid (CT; tensor of the vocal fold) muscle (Fig. 1B). Three mucosa samples (10 × 10 mm/each) were taken from laryngeal surface of the epiglottis (LSE), aryepiglottic fold (AEF) (Fig. 1C), and true vocal fold (TVF) (Fig. 1D). These regions of mucosa were chosen because they have rich sensory nerve terminals that are responsible for eliciting swallowing and laryngeal closure to protect airway during swallowing. The muscle and mucosa samples were frozen in isopentane cooled by dry ice and sectioned longitudinally (40 µm thick) and/or transversely (10 µm thick) on a cryostat (Reichert-Jung 1800, Mannheim, Germany) at − 25 °C. The sections were stored at − 80 °C until staining was performed.

### Staining Methods

The longitudinal nerve sections were immunostained for PAS to identify PAS-immunoreactive (PAS-ir) axons. The longitudinal muscle and mucosa sections were stained using immunohistochemical methods, including neurofilament (NF) staining to label all the axons and immunostaining for PAS to identify PAS-ir axons. The muscle cross-sections were stained with hematoxylin and eosin to examine myofiber morphology and immunostained to detect fiber type-grouping, atrophied fibers, and denervated myofibers, as described below.

#### NF Staining to Label All the Axons

**Fig. 2:**
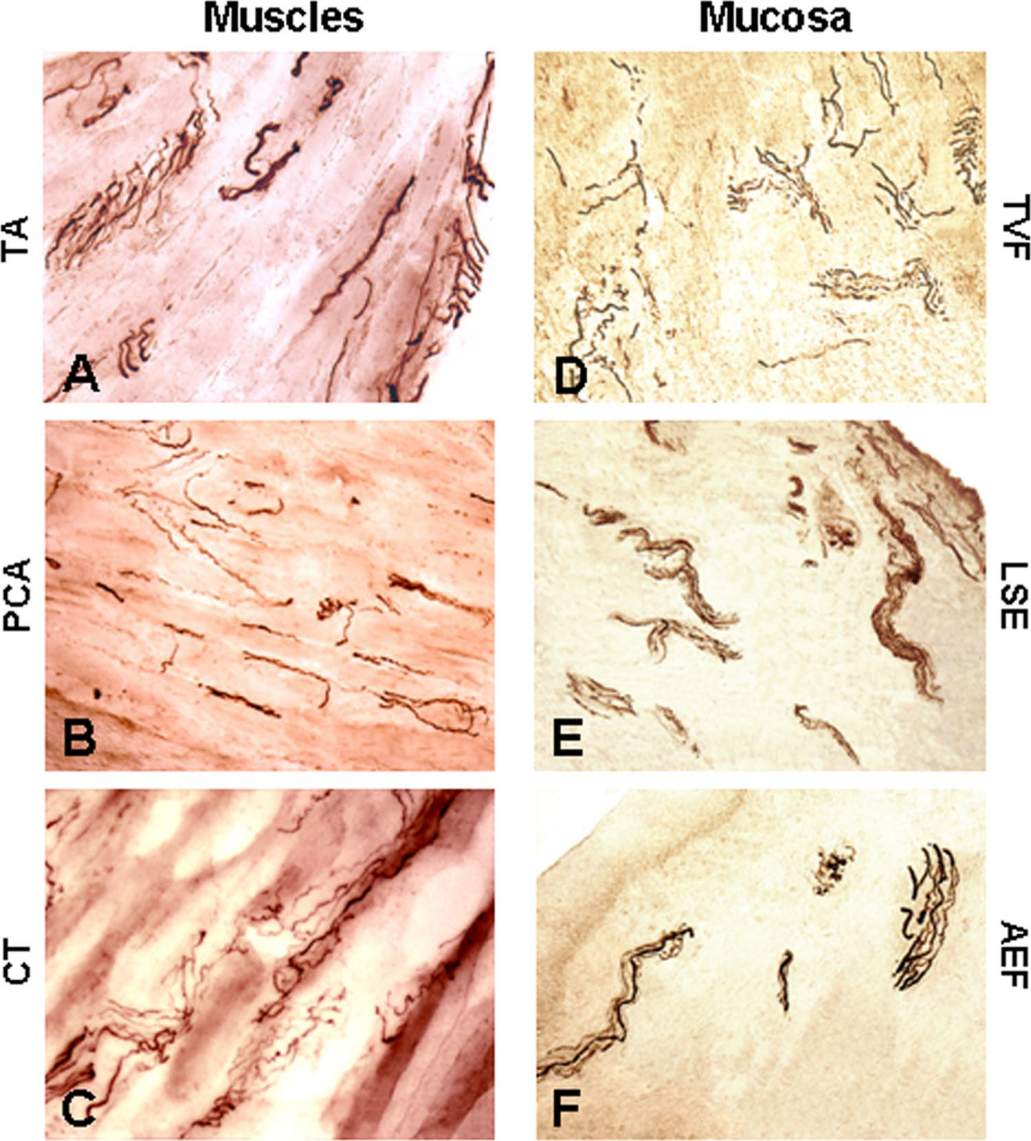
Photomicrographs of longitudinal sections of laryngeal muscles (**A–C**) and different regions of laryngeal mucosa (**D–F**) from a PD subject (PD no. 12). The sections were immunostained with monoclonal antibody SMI-31 against neurofilaments. Note that the RLN-innervated laryngeal muscles (i.e., TA, PCA and CT) and ISLN-innervated mucosa regions (i.e., TVF, LSE and AEF) examined have numerous intramuscular motor and intramucosal sensory nerve fascicles, twigs, and axons (dark staining). Magnification: 200 × for **A** through **F**

Some longitudinal muscle and mucosa sections were immunostained with monoclonal antibody SMI-31 against NF (Covance Research Products, Berkeley, CA) (Fig. 2) to label nerve fascicles, twigs, and axon terminals, as described [25, 38, 39]. Briefly, the sections were (1) blocked in phosphate-buffered saline (PBS) containing 0.3% Triton and 2% bovine serum albumin (BSA) for 30 min; (2) incubated with primary antibody SMI-31 (1:800; Covance Research Products, Berkeley, CA) in PBS containing 0.03% Triton at 4 ºC overnight; (3) incubated with anti-mouse biotinylated secondary antibody (1:1000; Vector Laboratories, Burlingame, CA) for 2 h; (4) treated with a VectaStain ABC kit (1:1000; ABC Elite, Vector); and (5) treated with diaminobenzidine-nickel as chromogen to visualize peroxidase labeling. Control sections were incubated without primary antibody.

#### PAS Immunohistochemistry

**Fig. 3:**
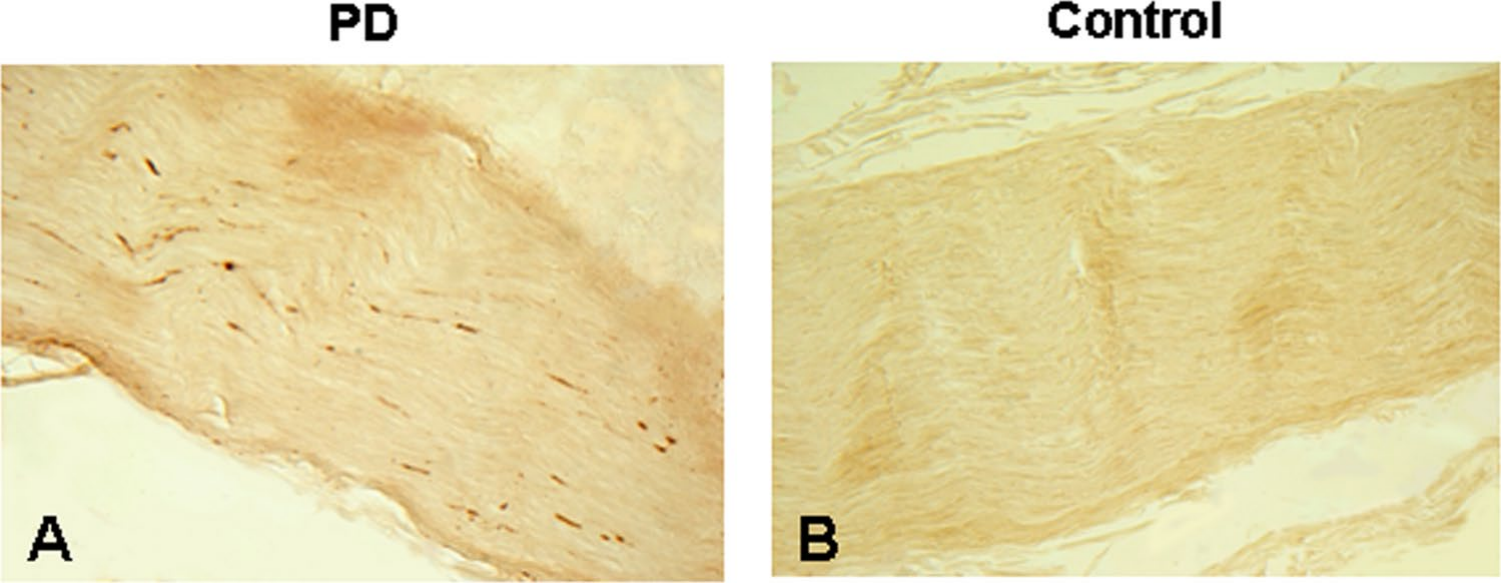
Photomicrographs of the longitudinal sections of the RLN from a PD patient (PD no. 5, male, age: 80) (**A**) and an age-matched normal control (NC no. 2, male, age: 80) (**B**). The sections were immunostained with monoclonal anti-phosphorylated α-synuclein antibody (psyn 64). Note that there are numerous PAS-ir axons (darkly stained threads and dots) in the RLN of the PD subject (**A**), whereas there are no PAS-ir axons in the control (**B**). Magnification: 200 × for **A** and **B**

**Fig. 4:**
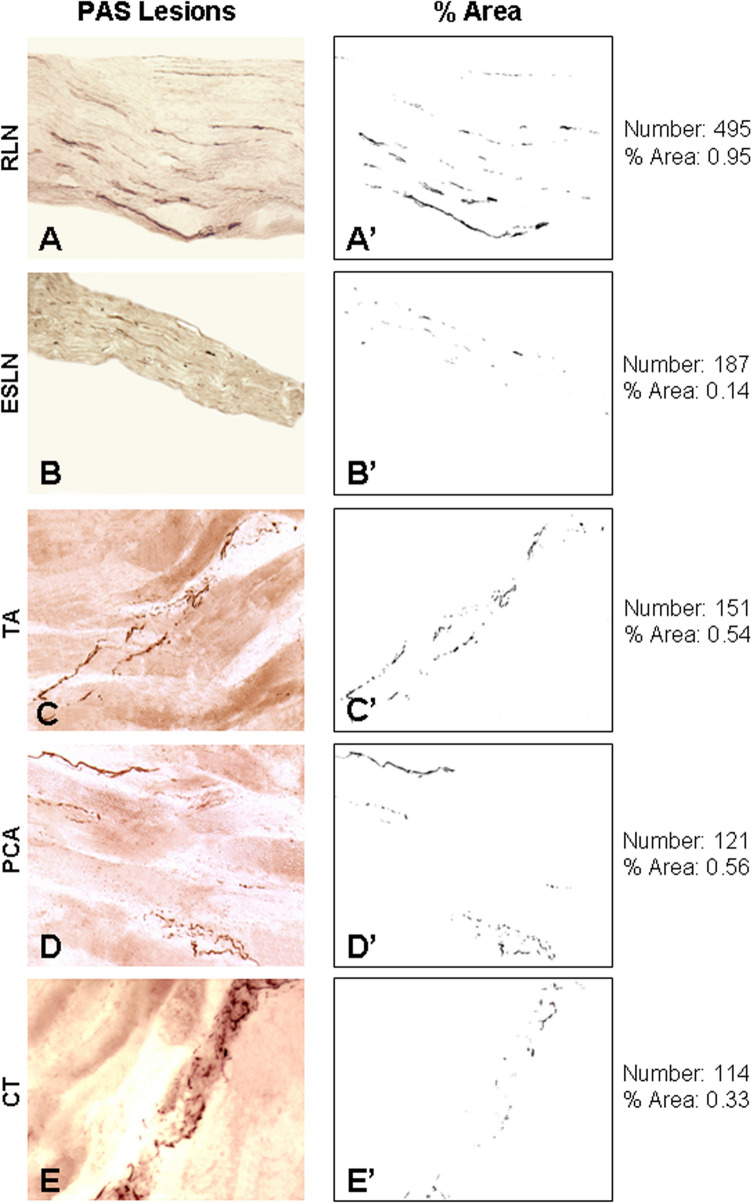
**A–E** Photomicrographs of the longitudinal sections of motor nerves (i.e., RLN and ESLN) (**A, B**) and their innervating muscles studied (i.e., TA, PCA, and CT) (**C–E**) from the same PD subject (PD no. 12) as that described in Fig. 2, who had severe SSV disorders (see Table 1). The sections were immunostained with monoclonal anti-phosphorylated α-synuclein antibody (psyn 64). Note that there are numerous PAS-ir axons (darkly stained threads and dots) in the laryngeal motor nerves and muscles. Magnification: 200 × for **A** through **E**. (**A’–E’**) The images in (**A–E**) were opened using ImageJ software and converted to 8-bit (binary) images, color thresholded, and particle analyzed to calculate the number and percent area of the PAS-ir axons in the peripheral laryngeal motor nervous system. For this subject, the mean number and mean area of PAS-ir axons in the laryngeal motor nerves and muscles examined were calculated to be 214 and 0.50, respectively

**Fig. 5:**
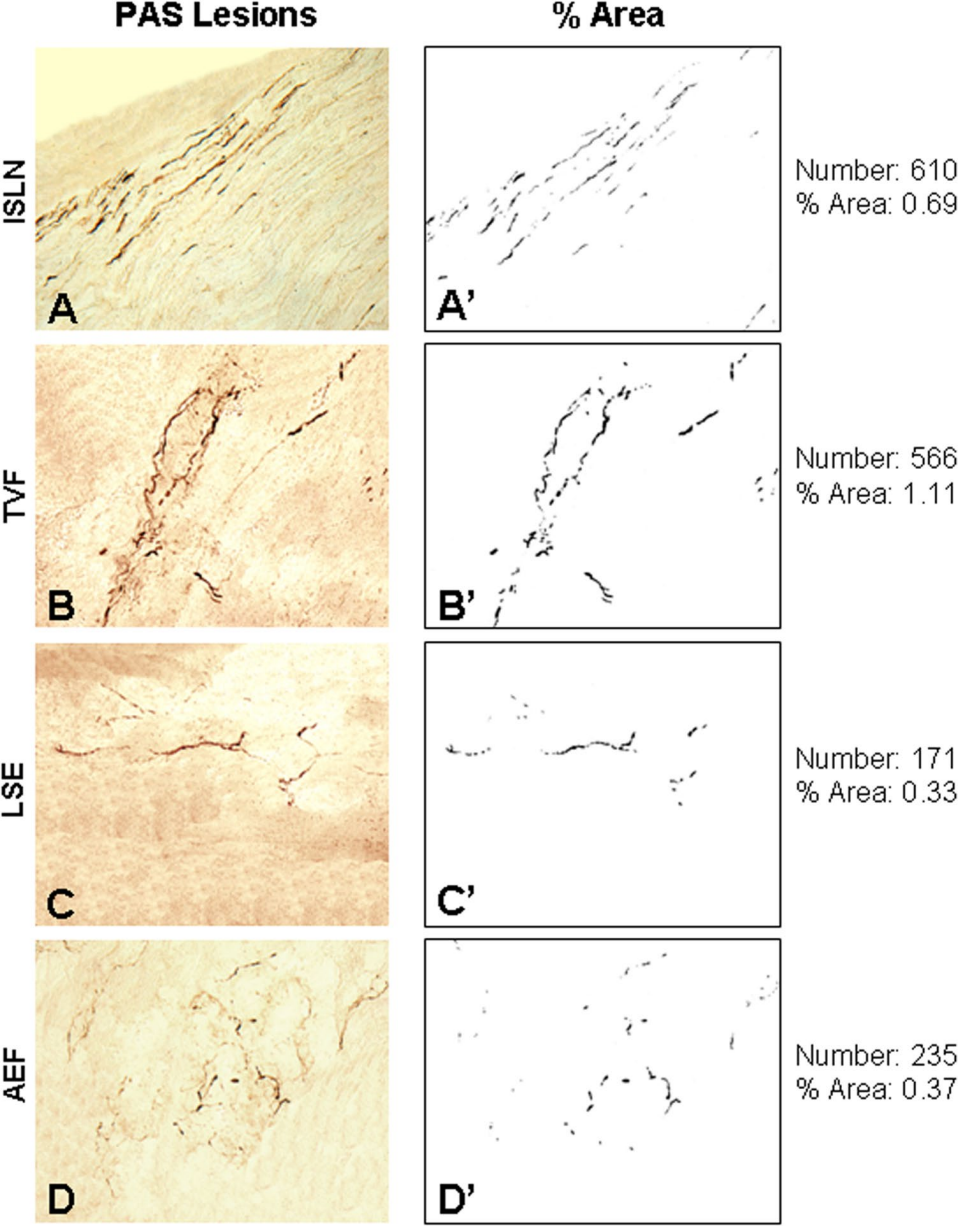
**A–D** Photomicrographs of the longitudinal sections of sensory nerve (i.e., ISLN) (**A**) and its innervating laryngeal mucosa (i.e., TVF, LSE, and AEF) (**B–D**) from the same PD subject (PD no. 12) as that described in Figs. 2 and 3. The sections were immunostained with monoclonal antibody psyn 64. Note that there are numerous PAS-ir axons (darkly stained threads and dots) in the ISLN and different mucosa regions examined. Magnification: 200 × for **A** through **D**. **A’–D’** The images in (**A–D)** were converted to 8-bit (binary) images by the use of ImageJ software to compute the number and percent area of the PAS-ir axons in the peripheral laryngeal sensory nervous system. For this subject, the mean number and mean area of PAS-ir axons in the ISLN and laryngeal mucosa regions were calculated to be 396 and 0.62, respectively

Some longitudinal sections of the laryngeal nerves, muscles and mucosa were immunostained for PAS (Figs. 3, 4, 5), as described [22–25, 40, 41]. In brief, the tissue sections were (1) pretreated with proteinase K (1:100; Enzo Life Sciences, Farmingdale, NY) diluted in 0.1 mol/L PBS at 37 °C for 30 min; (2) immersed in 1% H_2_O_2_ in 0.1 mol/L PBS with 0.3% Triton X-100 (PBS-TX) at pH 7.4 for 30 min; (3) incubated in anti-PAS monoclonal antibody (psyn no. 64; 1:1000; Wako, Richmond, VA) in PBS-TX at 4 °C overnight; (4) incubated in biotinylated anti-mouse IgG (1:000; VectaStain kit, Vector) in PBS-TX for 2 h at room temperature (RT); (5) treated with avidin–biotin complex (ABC; Vector), with A and B components of the kit both at 1:1000 dilution for 30 min; (6) treated with 3,3׳-diaminobenzidine (Sigma, St. Louis, MO) (5 mg/100 ml) with added saturated nickel ammonium sulfate (2/100 mL) and H_2_O_2_ (5 μL/100 mL of 1% H_2_O_2_) in the dark for 30 min. The treated sections were washed 3 times in PBS-TX between staining steps. Control sections were incubated without a primary antibody.

#### Immunostaining to Identify Fiber Type-Grouping and Atrophied Myofibers

**Fig. 6:**
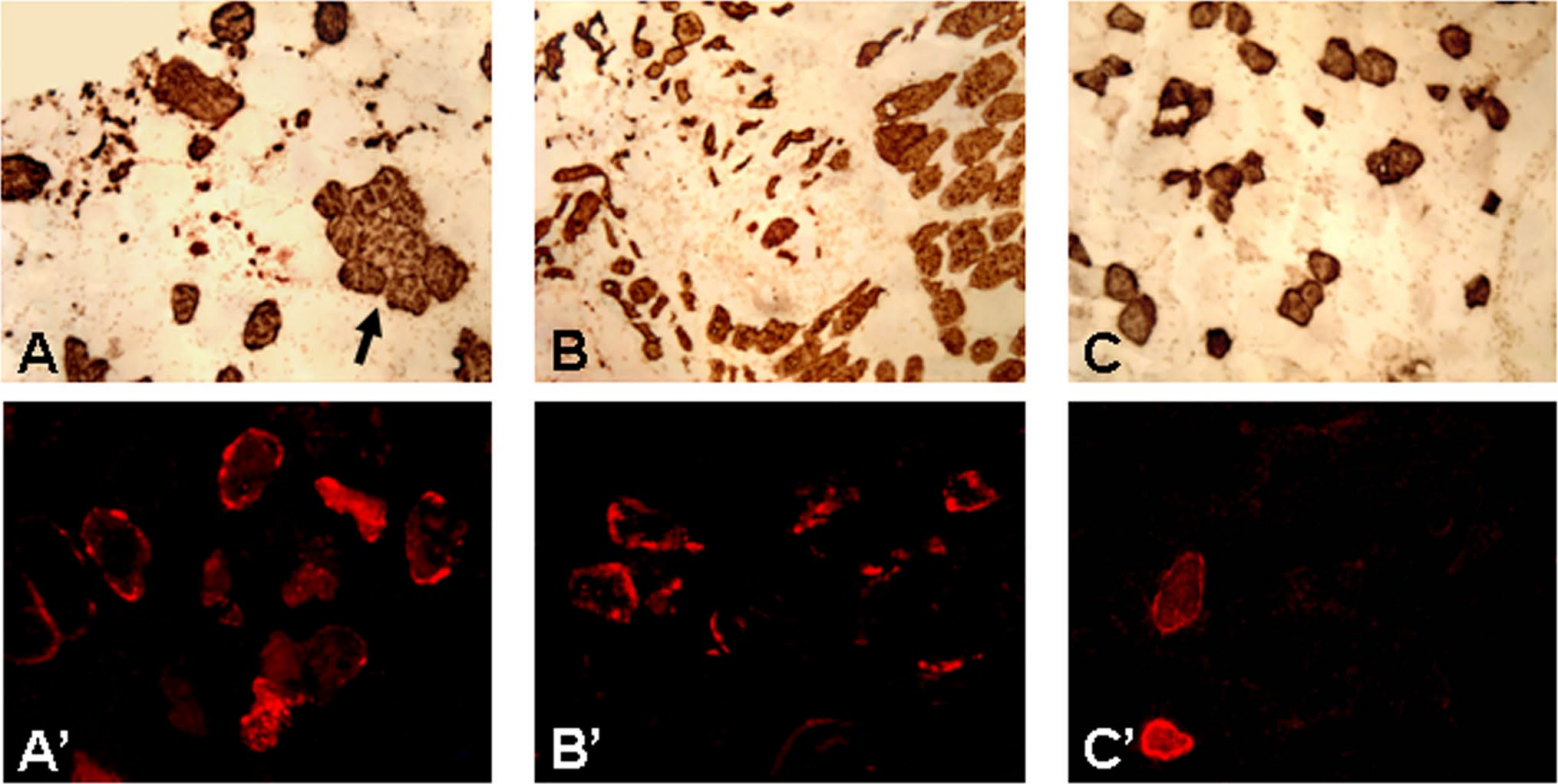
Photomicrographs of the cross-sections of the TA muscles from PD no. 12 (**A, A’**), PD no. 15 (**B, B’**), and NC no. 2 (**C, C’**). The sections were stained with monoclonal antibody NOQ7-5-4-D (**A–C**) to label slow type I fibers (dark staining) and stained for N-CAM (**A’–C’**) to identify denervated muscle fibers (bright staining). Fiber type-grouping (arrow in **A**) and atrophied myofibers (**A, B**) were identified in the TA muscle of the PD subjects. N-CAM immunostaining showed that there were more denervated muscle fibers in the PD muscles (**A’ B’**) than in the controls. There were a few atrophied and denervated myofibers in the control muscles (**C, C’**). Magnification: 100 × for **A**–**C** and 200 × for **A**’–**C**’

Some cross-sections of the laryngeal muscles examined were immunostained with monoclonal antibody NOQ7-5-4D to label slow type I muscle fibers for identifying fiber type-grouping and atrophied myofibers (Fig. 6A–C) caused by partial denervation, as described [24]. In brief, muscle sections were (1) fixed in 4% paraformaldehyde for 10 min; (2) blocked in 2% BSA and 0.1% Triton X-100 for 20 min; (3) incubated with monoclonal antibody NOQ7-5-4D (1:1000; Sigma, St. Louis, MO) for 1 h at RT; (4) incubated with an anti-mouse IgG (ATCC, Rockville, MD) for 1 h; (5) reacted in ABC reagent for 1 h; and (6) processed with DAB substrate kit (SK-4100; Vector) for 10 min. Control sections were stained without primary antibody.

#### Neural Cell Adhesion Molecule (N-CAM) Immunohistochemistry to Detect Denervated Myofibers

N-CAM is a molecular marker of muscle fiber denervation. It is abundant on the surface of early embryonic myotubes, decreases in level as development proceeds, almost disappears in the adult muscle, and reappears when adult muscles are denervated [42–44]. Therefore, N-CAM immunostaining is widely used for detecting denervated myofibers [44–47]. In this study, immunofluorescent labeling of N-CAM was performed (Fig. 6A’–C’) as described in our publications [24, 48]. Briefly, cross-sections of the laryngeal muscles were (1) fixed with methanol at − 20 °C for 20 min; (2) blocked with 5% donkey serum (Sigma) in PBS for 30 min; (3) incubated with a primary rabbit anti-rat N-CAM antibody (Chemicon, Temecula, CA) for 2 h; and (4) incubated with a secondary CY3-conjugated donkey anti-rabbit IgG (Jackson ImmunoResearch Laboratories, West Grove, PA) for 1 h. The sections were washed extensively in PBS between steps. Control sections were stained without primary antibody. The stained sections were mounted with Vectashield mounting medium (Vector Laboratories), kept in the dark at 4 °C, and photographed.

### Image Acquisition and Analysis

The stained sections of the laryngeal nerves, muscles and mucosa were examined under a Zeiss photomicroscope (Axioplan-1; Carl Zeiss, Gottingen, Germany) and photographed using a USB 3.0 digital microscope camera (Infinity 3-3URC; Lumenera Corp., Ottawa, Ontario, Canada). Images at a magnification of 200 × were imported into an image-processing program (ImageJ v. 1.45 s; National Institutes of Health, Bethesda, MD) to compute the number and area fraction of the PAS-ir axons within a section area (1.0 mm^2^).

### Assessments of PNS Pathology Severity

For a given tissue sample (i.e., laryngeal nerves, muscles, and mucosa), three sections at different spatial levels stained for PAS were examined to select one with the greatest number of PAS-ir axons. A microscopic field with the highest density of PAS-ir axons in the selected section was identified and photographed at 200 × magnification to estimate the number and area fraction of the PAS-ir axons with ImageJ.

On the basis of our prior studies on the PD pharynx [22, 23, 25], we developed a total PNS pathology score (TPPS) to indicate the PAS lesion severity. The TPPS consisted of a motor PAS score and a sensory PAS score. A motor PAS score was derived from the mean number of the PAS lesions (MNPL) in the motor nerves (i.e., RLN and ESLN) and their innervating muscles (i.e., TA, PCA, and CT). A sensory PAS score was derived from the MNPL in the sensory nerve (i.e., ISLN) and its innervating regions of the laryngeal mucosa (i.e., TVF, LSE, and AEF). For each PD subject, the motor and sensory PAS scores were summed and averaged to yield a TPPS, a measure of PAS pathology severity in the larynx by using a 4-tier scale (0–3): 0 = no lesions, 1 = 1 to 100 lesions (mild), 2 = 101 to 200 lesions (moderate), 3 = more than 200 lesions (severe) as calculated by ImageJ.

### Statistical Analyses

The data gathered in the study were analyzed using SAS 9.4 (SAS Institute Inc., Cary, NC, USA) or R software version 4.1.0 (R Foundation for Statistical Computing, Vienna, Austria). Continuous random variables were summarized as mean or median (interquartile range) depending on whether the data are from a normal distribution. Categorical variables were presented as count (percentages) and examined using *Fisher’s* exact test or *Pearson’s Chi-square* test, as appropriate. Correlation between swallowing and speech scores and disease severity levels (i.e., H&Y stages and motor UPDRS), PNS and CNS pathology severity levels (i.e., TPPS, TBS, and SNPNL) were evaluated using Spearman’s rank correlation. Scatterplots with Locally Estimated Scatterplot Smoothing (LOESS) fit were produced to depict the relationship between speech score, swallowing score, SS score (defined as the sum of swallowing score and speech score), and TPPS. LOESS is a non-parametric regression technique that fits a smooth curve to a set of data points correlation between both PD durations (shorter vs. longer) and H&Y stages (H&Y stages 2, 3 vs. 4, 5), along with age at PD onset and sex, against swallowing score, speech score, TPPS, TBS, and SNPNL were examined using ordinal logistic regression and simple logistic regression, as appropriate. Statistical significance was set at *p* < 0.05.

## Results

### Demographics

Table 1 summarizes the demographics and relevant clinical features of the PD and control subjects. There were 20 PD subjects (16 men and 4 women) with a mean age of 76 years (range, 65–84 years). All PD subjects were white. The mean age at PD onset was 61 years (range, 45–72 years; 12 subjects were > 60 years and 8 subjects ≤ 60 years). The mean duration of PD at the time of death was 15 years (range, 5–30 years). The mean last H&Y stage was 3.6 (range, 2–5). The mean last score for the motor UPDRS (UPDRS: Part III) was 42 points (range, 17–68). The mean interval between last neuro/movement examination and autopsy was 11 months (range, 1–28 months) for PD subjects. There were 8 age-matched normal control (NC) cases (4 men and 4 women), with a mean age of 76 years (range, 70–80 years).

### Severity of SSV Disorders

The UPDRS Part II Scale showed that 15 (75%) PD subjects reported signs/symptoms of dysphagia (Table 1). In the 15 dysphagic PD patients, 14 reported mild-to-moderate dysphagia (swallowing scores of 1 in 7 cases and 2 in 7 cases) and 1 reported severe dysphagia (swallowing score: 4). On the basis of the items 5 and 18 of the UPDRS parts II and III Scales, impaired speech and voice occurred in 18 (90%) out of the 20 subjects, 7 (39%) were mild-to-moderate (speech scores of 1 in 2 cases and 2 in 5 cases) and 11 (61%) were moderate-to-severe (speech scores of 3 in 7 cases and 4 in 4 cases). In the 20 PD subjects, 13 (65%) had co-occurrence of both dysphagia and impaired speech and voice.

Of the 20 PD subjects, 5 (PD no. 6, 10, 12, 16, 19) had objective SSV assessments, including MBS, fiberoptic flexible laryngoscopy, and/or laryngeal videostroboscopy. PD no. 12, with severe SSV deficits (swallowing score: 4; speech score: 4) (Table 1) had moderate-to-severe hypophonia, dysarthria, and imprecise articulation. MBS showed evidence of dysphagia and aspiration. PD no. 19 had moderate-to-severe SSV deficits (swallowing score: 2; speech score: 3) (Table 1). Laryngeal videostroboscopy showed that this patient had true vocal fold bowing, incomplete glottic closure, and reduced vibratory mucosal wave and amplitude of the vocal fold. Although some patients had very mild dysphagia (swallowing score: 1; speech score: 0), MBS showed hesitancy of bolus formation in the oral stage, persistent coating of vallecula, laryngeal penetration and aspiration.

### Brain Neuropathology

Table 1 summarizes the total brain score (TBS: 0–40) and the SNPNL score (0–3) for each PD subject. Specifically, the mean TBS (n = 17) was 31 (range, 24–37) and the mean SNPNL score (n = 19) was 2.7 (range, 2–3). The TBS showed that there was no case with mild PAS severity (TBS: 0–20); 10 (10/17; 59%) cases with moderate (TBS: 21–30) and 7 (7/17; 41%) cases with severe (TBS: 31–40) PAS pathology. The SNPNL scores showed that there was no subject with mild SNPNL (SNPNL: 1); 6 (6/19; 32%) with moderate (SNPNL: 2) and 13 (13/19; 68%) with severe (SNPNL: 3) SNPNL.

### PNS Pathology

The longitudinal sections stained with NF staining showed that there are numerous intramuscular motor (Fig. 2A–C) and intramucosal sensory (Fig. 2D–F) nerve fascicles, twigs, and axons. The high innervation density is indicative of the delicate neural control of the larynx.

In this series, PAS-ir axons were identified in all PD larynges, but not in the controls (Fig. 3). PAS-ir axons were present in the laryngeal motor nerves (i.e., RLN and ESLN) (Fig. 4A, B, A’, B’) and their innervating muscles studied (Fig. 4C–E, C’–E’) as well as sensory nerve (i.e., ISLN) (Fig. 5A, A’) and its innervating mucosa regions (i.e., TVF, LSE, and AEF) (Fig. 5B–D, B’–D’) with variable densities (Table 2).

**Table 2:** Distribution of PAS lesions in the motor and sensory components of the larynx in PD subjects

PD no.	MNPL (motor) (RLN, ESLN, TA, PCA, CT)	MNPL (sensory) (ISLN, TVF, LSE, AEF)	Average	TPPS
1	97	90	94	1
2	126	133	130	2
3	81	63	72	1
4	74	82	78	1
5	183	130	157	2
6	61	93	77	1
7	71	108	90	1
8	144	261	203	3
9	184	252	218	3
10	234	211	223	3
11	215	268	242	3
12	214	396	305	3
13	210	266	238	3
14	48	96	72	1
15	153	158	156	2
16	62	85	74	1
17	146	182	164	2
18	99	74	87	1
19	305	174	240	3
20	262	125	194	2
Average	**148**	**162**	**155**	**2.0**

AEF, aryepiglottic fold; CT, cricothyroid muscle; ESLN, external superior laryngeal nerve; ISLN, internal superior laryngeal nerve; LSE, laryngeal surface of epiglottis; MNPL, mean number of the PAS lesions; PAS, phosphorylated α-synuclin; PCA, posterior cricoarytenoid muscle; RLN, recurrent laryngeal nerve; TA, thyroarytenoid muscle; TPPS, total PNS pathology score; TVF, true vocal fold

Table 2 summarizes the distribution of PAS-ir axons in both the motor and sensory components in the larynx and the TPPS (TPPS: 0–3) for each PD subject. As demonstrated by TPPS, PNS pathology severity was rated as negative (TPPS: 0) in no case, mild (TPPS: 1) in 8 (40%) cases, moderate (TPPS: 2) in 5 (25%) cases, and severe (TPPS: 3) in 7 (35%) cases.

PAS lesions in the laryngeal motor nerves and muscles induced nerve degeneration that resulted in fiber type-grouping, myofiber denervation and atrophy. These muscle alterations were more predominant in the TA than the PCA and CT and identified mainly in slow type I fiber population (Fig. 6A, B). N-CAM immunostaining labeled denervated myofibers in the PD laryngeal muscles, especially in the TA muscle (Fig. 6A’, B’). There were a few atrophied and denervated myofibers in the control muscles (Fig. 6C, C’).

### Correlations of SSV Severity with Disease Duration and Severity, TPPS, and TBS/SNPNL

Table 1 summarizes the overall distribution of the PD duration, H&Y stage and motor UPDRS, swallowing score, speech score, TPPS, TBS and SNPNL score for each PD subject. The relationships between these parameters were analyzed.

In this series, the mean PD duration was 15 years (range, 5–30 years) (Table 1). There were 12 (60%) cases with a relatively shorter duration (SD; 0–15 years; mean: 9 years) and 8 (40%) with a longer duration (LD; 16–30 years; mean: 22 years) (Table 3).

**Table 3:** Correlations of PD duration (0–15 and 16–30 years) with SSV severity, TPPS, and TBS/SNPNL

PD no.	PD duration, years	Swallowing Score (0–4)	Speech Score (0–4)	TPPS (0–3)	TBS (0–40)	SNPNL Score (0–3)
	(0–15 years)					
2	11	0	3	2	37	3
5	11	0	3	2	37	2
6	11	1	0	1	30	3
7	11	0	2	1	36	2
11	10	2	4	3	36	2
12	5	4	4	3	24	3
14	6	1	0	1	27	3
15	15	1	3	2	29	3
17	5	1	2	2	27	3
18	6	1	2	1	29	3
19	12	2	3	3	30	3
20	7	2	3	2	36	3
Mean	**9**	**1.3**	**2.4**	**1.8**	**32**	**2.8**
	(16–30 years)					
1	20	1	2	1	35	3
3	19	2	2	1	–	–
4	20	0	1	1	26	3
8	30	2	4	3	35	3
9	17	0	3	3	–	2
10	23	2	3	3	26	2
13	21	1	4	3	28	3
16	29	2	1	1	–	2
Mean	**22**	**1.3**	**2.5**	**2.0**	**30**	**2.6**

PNS, peripheral nervous system; SNPNL, substantia nigra pigmented neuron loss; SSV, swallowing, speech and voice; TBS, total brain score; TPPS, total PNS pathology score

There were 8 (40%) PD subjects with earlier stages (H&Y ≤ 3) and 12 (60%) with later stages (H&Y > 3) (Table 4). For the earlier and later disease stages, the mean H&Y was 2.5 and 4.3 and the mean motor UPDRS was 28 and 52 points, respectively (Table 4).

**Table 4:** Correlations of PD severity (earlier and later H&Y stages) with SSV severity, TPPS, and TBS/SNPNL

PD no.	Last H&Y stages	Last motor UPDRS	Swallowing Score (0–4)	Speech Score (0–4)	TPPS (0–3)	TBS (0–40)	SNPNL Score (0–3)
	Earlier stages						
1	2	17	1	2	1	35	3
2	3	18	0	3	2	37	3
4	3	29	0	1	1	26	3
10	2	28	2	3	3	26	2
14	2	41	1	0	1	27	3
16	3	20	2	1	1	–	2
17	3	2	1	2	2	27	3
18	2	21	1	2	1	29	3
Mean	**2.5**	**28**	**1.0**	**1.8**	**1.4**	**30**	**2.8**
	Later stages						
3	4	51	2	2	1	–	–
5	4	53	0	3	2	37	2
6	4	43	1	0	1	30	3
7	4	47	0	2	1	36	2
8	4	66	2	4	3	35	3
9	5	40	0	3	3	–	2
11	4	55	2	4	3	36	2
12	5	54	4	4	3	24	3
13	4	66	1	4	3	28	3
15	4	45	1	3	2	29	3
19	4	31	2	3	3	30	3
20	5	68	2	3	2	36	3
Mean	**4.3**	**52**	**1.4**	**2.9**	**2.3**	**29**	**2.4**

H&Y stages, Hoehn and Yahr clinical rating scale (score range, 1–5); PNS, peripheral nervous system; SNPNL, substantia nigra pigmented neuron loss; SSV, swallowing, speech and voice; TBS, total brain score; TPPS, total PNS pathology score; UPDRS, Unified Parkinson’s disease rating scale

The correlations between both PD duration (i.e., SD vs. LD) and disease severity (i.e., H&Y stages 2–3 vs. 4–5) against swallowing score, speech score, TPPS, TBS, and SNPNL were analyzed using ordinal logistic regression and simple logistic regression, as appropriate. The results showed that there was no positive association of PD duration (SD vs. LD) with SSV severity, and PNS and CNS pathology severity (*p* > 0.05). However, there was a statistically significant association between speech score and H&Y later stages (i.e., stages 4–5) (*p* = 0.0230). The relationship between TPPS and H&Y later stages was reported as trending towards significance (*p* = 0.0642, proportionality assumption met). These results indicate that a larger sample size is warranted to uncover possible significant associations.

The relative contributions of PNS and CNS pathologies to SSV disorders were estimated by analyzing the associations between the parameters examined. In this series, the SSV severity levels in 9 (45%) PD subjects (PD no. 1, 2, 4, 6, 7, 10, 14, 18, 20) were consistent more with PNS (i.e., TPPS) than CNS (i.e., TBS and SNPNL) pathology severity levels (Table 5, top panel). For example, some PD cases (PD no. 4, 6, 14) with very mild SSV deficits (swallowing score: 0, 1, 1; and speech score: 1, 0, 0, respectively) had mild PNS pathology (TPPS: 1, 1, 1, respectively) but moderate-to-severe CNS pathology (TBS/SNPNL: 26/3, 30/3, 27/3, respectively). Some cases such as PD 6 with severe disease severity (H&Y: 4; motor UPDRS: 43 points) and moderate-to-severe CNS pathology (TBS: 30; SNPNL: 3) had very mild SSV deficits (swallowing score: 1; speech score: 0) and mild PNS pathology (TPPS: 1) (Table 4). These findings indicate that PNS pathology levels of these PD subjects correlate well with their SSV severity levels, but not with CNS pathology levels.

**Table 5:** Correlations of SSV severity (swallowing and speech scores) with levels of PNS (TPPS) and CNS (TBS and SNPNL) pathologies

PD no.	Swallowing Score (0–4)	Speech Score (0–4)	TPPS (0–3)	TBS (0–40)	SNPNL Score (0–3)
SSV severity levels were consistent more with PNS than CNS pathology severity levels					
1	1	2	1	35	3
2	0	3	2	37	3
4	0	1	1	26	3
6	1	0	1	30	3
7	0	2	1	36	2
10	2	3	3	26	2
14	1	0	1	27	3
18	1	2	1	29	3
20	2	3	2	36	3
Mean	**0.9**	**1.8**	**1.4**	**31**	**2.8**
SSV severity levels were consistent with both the PNS and CNS pathology severity levels					
3	2	2	1	–	–
5	0	3	2	37	2
8	2	4	3	35	3
9	0	3	3	–	2
11	2	4	3	36	2
12	4	4	3	24	3
13	1	4	3	28	3
15	1	3	2	29	3
16	2	1	1	–	2
17	1	2	2	27	3
19	2	3	3	30	3
Mean	**1.5**	**3.0**	**2.4**	**31**	**2.6**

CNS, central nervous system; PNS, peripheral nervous system; SNPNL, substantia nigra pigmented neuron loss; SSV, swallowing, speech and voice; TBS, total brain score; TPPS, total PNS pathology score

In 11(55%) PD subjects (PD no. 3, 5, 8, 9, 11–13, 15–17, 19), their SSV severity levels were generally consistent with both the PNS and CNS pathology severity levels (Table 5, bottom panel). For example, PD no. 8, 12, 19 with severe disease (H&Y: 4, 5, 4; motor UPDRS: 66, 54, 31, respectively) and moderate-to-severe SSV deficits (swallowing score: 2, 4, 2; speech score: 4, 4, 3, respectively) had severe PNS pathology (TPPS: 3, 3, 3, respectively) and moderate-to-severe CNS pathology (TBS/SNPNL: 35/3, 24/3, 30/3, respectively) (Table 4). These findings suggest that comparable levels of both the PNS and CNS pathologies contributed to SSV disorders in these PD subjects.

The correlations between the severity of SSV disorders (i.e., swallowing score, speech score, and SS score) and PD pathology severity in the PNS and CNS (i.e., TPPS, TBS and SNPNL) were evaluated using Spearman’s rank correlation analysis. The results indicate that speech score was significantly correlated with TPPS (Spearman’s r = 0.89, 95% CI 0.74 to 0.96, *p* < 0.0001), whereas there is no significant correlation between swallowing score and TPPS (Spearman’s r = 0.34, 95% CI -0.12 to 0.68, *p* = 0.1471). This is most likely due to the fact that the majority (12/20; 60%) of the PD patients in this series had no or mild dysphagia (swallowing score: 0 in 5 cases and 1 in 7 cases) (Table 5). The correlation between SS score (i.e., the sum of swallowing score and speech score) and TPPS was significantly positive (Spearman’s r = 0.78; 95% CI 0.51 to 0.91, *p* < 0.0001). These results indicate that the strong relationship between SS score vs TPPS was driven mainly by the speech score component of SS score. Correlation analysis also showed that swallowing score, speech score, and SS score were not significantly correlated with TBS (0.9605, 0.5648 and 0.2824, respectively) and SNPNL (1.000, 0.7639 and 0.6944, respectively).

The results of the correlation analyses as described above provide evidence to support our hypothesis that SSV disorders in PD are caused not only by nigrostriatal dopamine deficiency but also by PAS pathology in the PNS controlling the structures in the upper aerodigestive tract, including the larynx.

## Discussion

Although the majority of PD patients have SSV disorders, to the best of our knowledge, this is the first study to demonstrate PAS pathology in the PNS controlling the larynx in PD. There are several key findings. First, the larynx was affected by PD pathology as indicated by the presence of PAS lesions in the laryngeal nerves, muscles, and mucosa. Second, nerve degeneration-induced muscle alterations (i.e., fiber type-grouping, denervated and atrophied myofibers) were identified mainly in the TA, followed by the PCA and CT muscles. Third, PNS pathology severity in the PD larynx was assessed by using our newly developed TPPS. Finally, our data showed that self-reported SSV severity levels in a substantial proportion (45%) of PD subjects were more consistent with PNS than brain pathology severity levels. These findings suggest that PNS α-synuclein pathology contributes to the severity of SSV disorders in PD.

SSV disorders in PD result from a combination of motor and nonmotor deficits. PAS pathology in the PNS of the larynx in PD could be an underlying cause of laryngeal dysfunction leading to dysphonia, dysphagia, aspiration, and aspiration pneumonia. As reported, the majority of PD patients have incomplete, delayed or absent reflex glottic closure during swallowing [16, 49]. It is known that sensory receptors in the laryngeal and laryngopharyngeal mucosa innervated by the ISLN are responsible for eliciting swallowing [50], reflex glottic closure [51, 52], and coughing [53, 54]. ISLN sensory dysfunction is attributed to dysphagia, incomplete laryngeal closure, aspiration, and impaired cough reflex [55, 56]. Recent studies by Hegland and colleagues [7, 57–59] have demonstrated decreased cough sensitivity and aspiration in individuals with PD. Dysphagia and cough dysfunction can lead to laryngeal penetration and aspiration pneumonia, a leading cause of death in PD patients [6, 8, 9]. The PAS lesions in the ISLN and its innervating mucosa in PD patients could result in sensory nerve degeneration, contributing to decreased laryngeal and laryngopharyngeal sensitivity as demonstrated by using fiberoptic endoscopic evaluation of swallowing with sensory testing developed by Aviv and colleagues [60–63]. PD patients with dysphagia often exhibited abnormal airway somatosensory function [64]. As reported, approximately 75% of the dysphagic individuals have severe laryngopharyngeal sensory deficits which lead to laryngeal penetration and aspiration [62].

Voice and speech disorders affect up to 90% of people with PD during the course of their disease. PD patients often have hypophonia characterized by a harsh, hoarse, weak or breathy voice quality [1–3]. Hypophonia is caused mainly by incomplete glottic closure due to vocal fold bowing (VFB) that has been identified in 87–94% of the PD patients [65–70]. More recently, a positive association between VFB severity and dysphonia severity in PD was revealed by quantitative measurements of vocal fold, glottal gap area, and voice [71]. However, the pathophysiology of VFB in PD remains unknown. The present study provided direct histopathological evidence for the clinically observed vocal fold atrophy and VFB. The cause of the VFB would be PAS lesions in the laryngeal motor nerves and muscles that result in nerve degeneration, muscle denervation, myofiber atrophy, and impaired vocal fold movements. Vocal fold atrophy causing VFB with a midfold glottic gap when voicing during speech is associated with a loss of air and reduced voice intensity during phonation, thereby leading to dysphonia. Thus, VFB is a potential risk factor leading to dysphonia and dysphagia in PD. Voice and speech disorders significantly impact communication and reduce quality of life for patients with PD if not addressed [72].

The present study showed that there was a positive correlation between the levels of SSV severity and PNS pathology severity in a substantial percentage of PD patients. Our prior work [22–25] and the present study indicate that PAS pathology in the PNS controlling the pharynx and larynx plays an important role in SSV disorders in PD. This may explain why SSV disorders in some PD patients do not respond well with anti-PD therapies that significantly improve limb motor symptoms. Current treatment modalities for PD-related SSV disorders including pharmacotherapy, surgical procedures, and behavioral speech therapy have variable effects on SSV deficits in PD [for review, see 73, 74]. Our findings may help facilitate the identification of peripheral therapeutic targets (i.e., nerves, mucosa regions, and muscles) in the structures of the upper aerodigestive tract for the development of novel therapies to treat PD-related SSV disorders.

## Limitations

The present study has a few limitations. Firstly, the severity of SSV disorders in PD was assessed mainly by UPDRS ratings (i.e., swallowing and speech scores). Objective evaluations, including imaging, for SSV deficits would be helpful for analyzing the correlations between SSV impairment severity and the severity of PNS and CNS α-synuclein pathology. Secondly, the sample size for this study was small. Therefore, the outcomes might not be representative for PD patients in general. Lastly, reliability of the TPPS for global severity ratings of PNS pathology was determined only in a small sample size. Further studies using a larger sample size are needed to validate this scoring system.

## Conclusions and Future Research Directions

This study provides a thorough and hypothesis-driven first look at the PD larynx. Our findings demonstrated PAS lesions in the laryngeal motor and sensory nerves and their innervating muscles and mucosa. In this study, we used our newly developed TPPS for evaluating the severity of PNS pathology in the PD larynx. The relative contributions of PNS and CNS pathologies to SSV disorders in PD were estimated. Correlation analyses provide strong evidence to support our hypothesis that SSV disorders in PD are caused by PAS pathology in both the CNS and PNS of the upper aerodigestive tract, including the larynx. Our newly developed TPPS scoring system would be a useful tool for quantifying global PNS pathology severity in PD. Further studies are needed to optimize and validate this scoring system for its use in the evaluation of PNS pathology severity.

## Data Availability

The data that support the findings of this study are available through the corresponding authors upon reasonable request.

## Abbreviations

ABC: Avidin-biotin complex
AEF: Aryepiglottic fold
AZSAND: Arizona Study of Aging and Neurodegenerative Disorders
BBDP: Brain and Body Donation Program
BSA: Bovine serum albumin
CNS: Central nervous system
CT: Cricothyroid muscle
ESLN: External superior laryngeal nerve
H&Y stages: Hoehn and Yahr clinical Parkinson’s disease rating scale
ISLN: Internal superior laryngeal nerve
LD: Longer duration
LOESS: Locally Estimated Scatterplot Smoothing
LSE: Laryngeal surface of the epiglottis
MBS: Modified barium swallow
MNPL: Mean number of PAS lesions
NC: Normal control
N-CAM: Neural cell adhesion molecule
NF: Neurofilament
PAS: Phosphorylated α-synuclein
PAS-ir: PAS-immunoreactive
PBS: Phosphate-buffered saline
PCA: Posterior cricoarytenoid muscle
PD: Parkinson’s disease
PMI: Postmortem interval
PNS: Peripheral nervous system
RLN: Recurrent laryngeal nerve
SD: Shorter duration
SLN: Superior laryngeal nerve
SNPNL: Substantia nigra pigmented neuron loss
SS score: Swallowing and speech score
SSV: Swallowing, speech and voice
TA: Thyroarytenoid muscle
TBS: Total brain score
TPPS: Total PNS pathology score
TVF: True vocal fold
UPDRS: Unified Parkinson’s Disease Rating Scale
USSLB: Unified staging system for Lewy body disorders
VFB: Vocal fold bowing
